# Haploinsufficiency of *KMT2D* is sufficient to cause Kabuki syndrome and is compatible with life

**DOI:** 10.1002/mgg3.1072

**Published:** 2019-12-08

**Authors:** Teresa Romeo Luperchio, Carolyn D. Applegate, Olaf Bodamer, Hans Tomas Bjornsson

**Affiliations:** ^1^ McKusick‐Nathans Department of Genetic Medicine Johns Hopkins University School of Medicine Baltimore MD USA; ^2^ Division of Genetics and Genomics Department of Pediatrics Boston Children's Hospital Harvard Medical School Boston MA USA; ^3^ Broad Institute of MIT and Harvard University Cambridge MA USA; ^4^ Department of Pediatrics Johns Hopkins University School of Medicine Baltimore MD USA; ^5^ Faculty of Medicine School of Health Sciences University of Iceland Reykjavik Iceland; ^6^ Landspitali University Hospital Reykjavik Iceland

## Abstract

We present the first patient described with haploinsufficency of *KMT2D* leading to Kabuki syndrome. Deletion of *KMT2D* has been thought to be lethal, but here we describe a patient with *KMT2D* deletion and classical Kabuki syndrome phenotype.

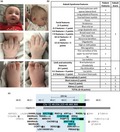

Dear Editor,

Kabuki syndrome (KS), also known as Niikawa‐Kuroki syndrome, is a Mendelian disorder of the epigenetic machinery which occurs in approximately 1:32,000 births characterized by intellectual disability, facial and limb dysmorphic features, and postnatal growth retardation. Pathogenic variants in genes *KMT2D* and *KDM6A* are found in 70% of patients with KS (type 1 MIM#:147920 and type 2 MIM#:300867 respectively). *KMT2D and KDM6A* are both highly constrained genes (pLI = 1.0) suggesting that both genes are intolerant to heterozygous loss‐of‐function variation and thus haploinsufficient. While true haploinsuffiency through deletion of the entire locus of *KDM6A* is a well‐established cause of KS2 (Lederer et al., 2012), no patient has yet to be described with a germline deletion of the entire *KMT2D* gene and it has been hypothesized that constitutional deletions of *KMT2D* may be embryonic lethal in humans (Banka et al., 2013). Despite this, *KMT2D* is classified in ClinGen as having sufficient evidence for haploinsufficiency based on the large number of truncating mutations that are causative of KS (dbVar: nsv997197/nssv3442621). Most molecularly confirmed cases of KS are from heterozygous pathogenic variants in *KMT2D* (94% *n* = 621/660) but in published reports only six cases (~0.9% *n* = 6/621) involve any type of *KMT2D* deletion larger than 20 basepairs (Bögershausen et al., 2016). Five of these involve small intragenic deletions and the sixth case is a mosaic whole gene deletion in a patient (Banka et al., 2013; Bögershausen et al., 2016). While it is assumed that mutations in *KMT2D* are mainly loss of function mutations, it has not been clear whether this gene is truly dosage sensitive.

Here, we describe the first published patient with a constitutional deletion of *KMT2D* with classical KS. Our patient is the first child of healthy parents born at term with a 2‐vessel cord and intrauterine growth restriction. She required intervention for hypothermia and feeding intolerance and was subsequently admitted to the pediatric intensive care unit (PICU) for hyperinsulinemic hypoglycemia. At 10 months of age, she is G‐tube fed with persistent hyperinsulinemic hypoglycemia requiring diazoxide and continuous overnight feeds, a phenotype seen in other children with KS1. She is hypotonic (trunk > extremities), with left hip dysplasia, joint laxity, and premature thelarche, all features previously seen in KS1. She has moderate bilateral hearing loss without a history of ear infections. An echocardiogram revealed a small‐medium atrial septal defect and a renal ultrasound was normal. She has the characteristic KS facial gestalt including elongated palpebral fissures, long eyelashes, arched eyebrows with sparseness of the lateral third of the brow, ptosis, a broad nasal root, flat nasal tip, high arched palate, micrognathia, and large cup‐shaped ears with an abnormally‐formed right ear pinna with pits (Figure 1a). The patient's mother also reports nocturnal lagophthalmos, a feature frequently reported in KS1. Our patient exhibits other distinctive physical features that are common among individuals with KS, including persistence of the fetal fat pads, and short 5th fingers with clinodactyly bilaterally (Figure 1a). She also has mild 2‐3 toe syndactyly, hypoplastic nails of hands and feet, and fused labia. As is seen in many children with KS, our patient demonstrates developmental delay. At 10 months she is cooing and smiling but not babbling, sits unassisted and has begun to pull to stand. Recently a phenotypic score has been validated for which the median score for molecularly confirmed KS is 6/10 (Makrythanasis et al., 2013). Our patient scores 5 on this system indicating that she objectively has a phenotype that fits KS very well (Figure 1b).

**Figure 1:**
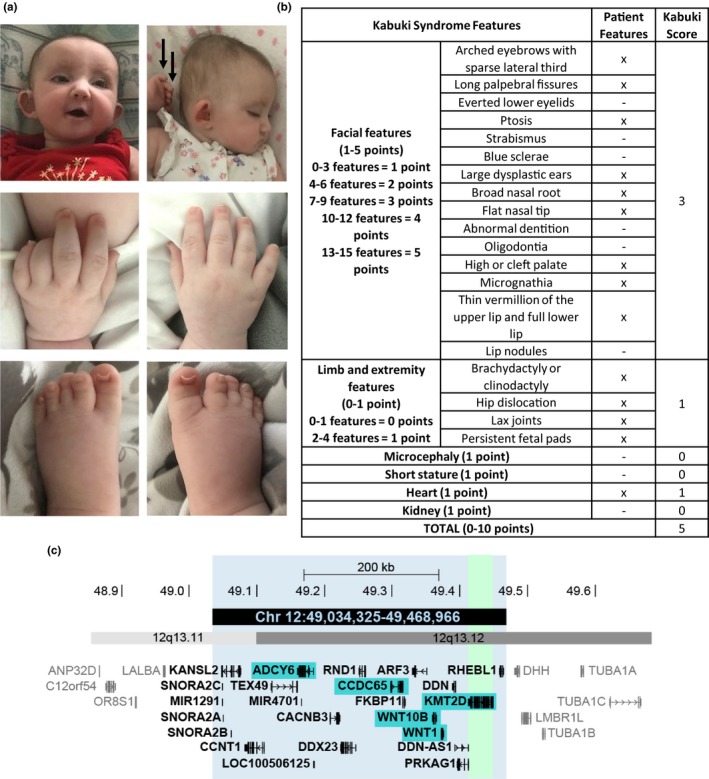
Phenotype of our patient with *KMT2D* haploinsufficiency. (a) Photos taken at 10 months show typical facial features and hand abnormalities such as persistent fetal fat pads (black arrows). (b) Our patient has a phenotype score of 5/10. (c) The 432 kb region which is deleted in our patient (black). Vertical green bar shows location of *KMT2D*. Genes highlighted in cyan are associated with an OMIM disease phenotype

Microarray analysis revealed a 434.6 kb deletion of 12q13.1 (Figure 1c). This deletion on chromosome 12 from 49,034,325 to 49,468,966 (hg19, ClinVar accession SCV000920777) encompasses 23 genes, five of which are genes associated with disease in OMIM (*ADCY6*, *CCDC65*, *WNT10B*, *WNT1*, *KMT2D*). Of these, only three diseases are inherited in an autosomal dominant pattern, Selective Tooth Agenesis‐8 (*WNT10B* MIM#:617073), susceptibility to early onset osteoporosis (*WNT1* MIM#:615221) and KS (*KMT2D* MIM#:602113). At 10 months, our patient has seemingly normal dentition with eruption of three normally formed teeth, and there have been no issues suggesting abnormal bone density. Our patient is the first known case to date of a nonmosaic whole‐gene deletion of *KMT2D* and presents with the typical clinical phenotype of KS. Early lethality due to haploinsufficency of *KMT2D* may be supported by the fact that on our evaluation of DECIPHER deletions and duplications that include all of *KMT2D*, there is marked overrepresentation of duplications (11/12), with the only deletion being very large (101.3 Mb). Though we cannot rule out reduced viability of pregnancies with a *KMT2D* deletion, deletion of *KMT2D* does not appear to be embryonic lethal as our patient is alive and well. Additionally, one would expect our patient to have a more severe clinical presentation than the average individual with KS if heterozygous deletion of *KMT2D* were to cause embryonic or in utero lethality; however, our patient's clinical presentation is no more severe than the average Kabuki patient, and from a congenital anomaly standpoint, her malformations are on the milder end of the spectrum.

Our patient is therefore the first described case of constitutional whole‐gene deletion of *KMT2D* in a patient with classical features of KS. The relatively small size of the deletion identified leads us to conclude that the loss of *KMT2D* is the principal cause of her clinical phenotype. This supports the notion that a primary cause of KS is decreased amounts of KMT2D protein rather than altered function which can be seen with gain‐of‐function or dominant‐negative mutations.

## CONFLICT OF INTEREST

HTB is a consultant for Millennium Pharmaceuticals.

## Data Availability

The variant presented in this manuscript has been submitted to ClinVar (accession SCV000920777).

## References

[mgg31072-bib-0001] Banka, S. , Howard, E. , Bunstone, S. , Chandler, K. E. , Kerr, B. , Lachlan, K. , … Donnai, D. (2013). MLL2 mosaic mutations and intragenic deletion‐duplications in patients with Kabuki syndrome. Clinical Genetics, 83(5), 467–471. 10.1111/j.1399-0004.2012.01955.x 22901312

[mgg31072-bib-0002] Bögershausen, N. , Gatinois, V. , Riehmer, V. Kayserili, H. , Becker, J. , Thoenes, M. , … Wollnik, B. (2016). Mutation update for kabuki syndrome genes KMT2D and KDM6A and further delineation of X‐linked kabuki syndrome subtype 2. Human Mutation, 37(9), 847–864. 10.1002/humu.23026 27302555

[mgg31072-bib-0003] Lederer, D. , Grisart, B. , Digilio, M. C. , Benoit, V. , Crespin, M. , Ghariani, S. C. , … Verellen‐Dumoulin, C. (2012). Deletion of KDM6A, a histone demethylase interacting with MLL2, in three patients with Kabuki syndrome. American Journal of Human Genetics, 90(1), 119–124. 10.1016/j.ajhg.2011.11.021 22197486 PMC3257878

[mgg31072-bib-0004] Makrythanasis, P. , van Bon, B. W. , Steehouwer, M. , Rodríguez‐Santiago, B. , Simpson, M. , Dias, P. , … Hoischen, A. (2013). MLL2 mutation detection in 86 patients with Kabuki syndrome: A genotype‐phenotype study. Clinical Genetics, 84(6), 539–545. 10.1111/cge.12081 23320472

